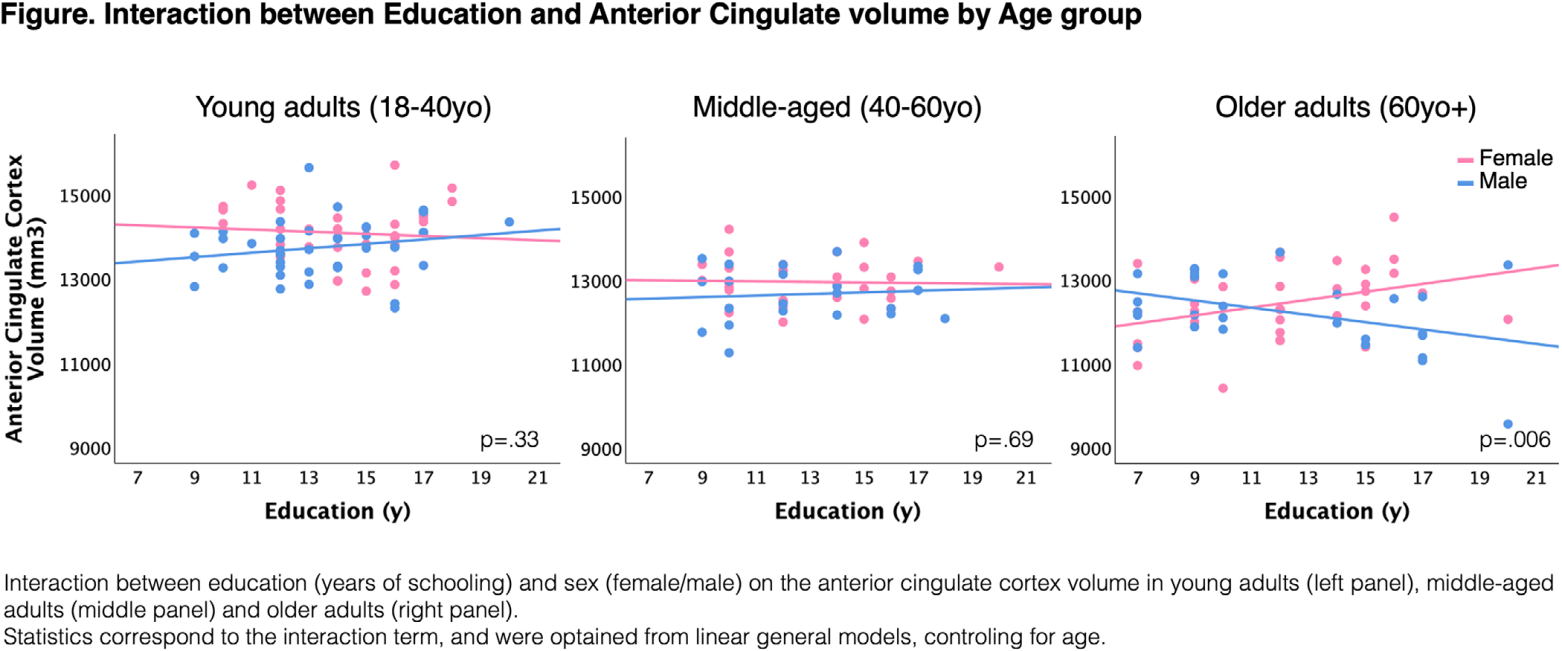# Association between education and multimodal neuroimaging in men and women across the lifespan

**DOI:** 10.1002/alz.094381

**Published:** 2025-01-09

**Authors:** Julie Gonneaud, Anne‐Laure TURPIN, Denis Vivien, Vincent De la Sayette, Gael Chételat

**Affiliations:** ^1^ Normandie Univ, UNICAEN, INSERM, U1237, PhIND “”Physiopathology and Imaging of Neurological Disorders“”, NeuroPresage Team, GIP Cyceron, Caen France; ^2^ Département de Recherche Clinique, CHU Caen‐Normandie, Caen France; ^3^ Inserm‐EPHE‐UNICAEN U1077, CAEN France

## Abstract

**Background:**

Education has been associated with reserve mechanisms and lower dementia risk, but the literature shows inconsistent results on the association between education and brain outcomes across the lifespan. Considering that both dementia risk and education are likely to differ between sexes, our study aims at understanding the association between education and brain outcomes across the lifespan and whether it differs by sex.

**Method:**

In 207 healthy individuals (110women) aged 19‐84 years old (47.98±18.75), we investigated the association between years of education and multimodal neuroimaging (structural‐MRI, FDG‐PET, Florbetapir‐PET) and how this association was modulated by age and sex. Analyses were restricted to regions involved in Alzheimer’s disease and/or reserve mechanisms (hippocampal volume, temporoparietal metabolism, neocortical amyloid, anterior and posterior cingulate cortices [ACC/PCC] for each modality).

**Result:**

There was no main effect of years of education on neuroimaging (ps>.09) nor interaction between education and the other variables. However, exploratory interactions conducted within young (18‐40), middle‐aged (40‐60) and older (60+yo) groups separately showed that, only in the older group, sex interacted with education on ACC volume, revealing that education was associated with greater volume only in older women (Figure; pinteraction = .006). Complementary analyses conducted in an independent and larger sample of older adults (n = 135, 83women, >65yo) in whom lifestyle was assessed retrospectively, suggest that midlife and late‐late‐life mental engagement, more than early‐life engagement (reflecting mainly education), are related to greater brain outcomes in late‐life (ACC volume, PCC amyloid burden, ACC metabolism). We assessed the moderative effect of sex on these associations and found that the association between grey matter volume and occupation (midlife engagement) was mainly driven by women (pinteraction = .02). The interaction between early‐life engagement and sex on ACC volume highlighted in the main cohort was replicated, yet at a trend‐level (pinteraction = .08).

**Conclusion:**

Our study suggests that education is not strongly associated with age‐related differences in structure, metabolism or amyloid burden. Older women with higher levels of education, however, showed higher ACC volumes. These results suggest that education could promote brain reserve in late life in a sex‐dependent manner. Future studies should investigate the mechanisms behind these differences in brain reserve.